# A School‐Based Nutrition and Physical Activity Intervention for the Prevention of Childhood Obesity: Study Design and Baseline Findings From a Cluster‐Randomized Trial

**DOI:** 10.1155/jnme/1016106

**Published:** 2026-05-31

**Authors:** Yeray Nóvoa-Medina, Sara del Cristo Leon, Marta Barreiro-Bautista, Verónica Batista-Dávila, Himar Fabelo, Svetlana Pavlovic, Yaiza García-Delgado, Juan Eugenio Jiménez, Sara López-López, Gran Canarian Diabetes and Obesity Research Group, Ana María Wägner, Luis Peña-Quintana

**Affiliations:** ^1^ Pediatric Endocrinology Unit, Insular-Maternal and Child University Hospital Complex of Las Palmas de Gran Canaria, Las Palmas de Gran Canaria, Spain; ^2^ Canarian Society for Pediatric Research (ACIP Canarias), Las Palmas de Gran Canaria, Spain; ^3^ University Institute of Biomedical and Healthcare Research of the University of Las Palmas de Gran Canaria, Las Palmas de Gran Canaria, Spain; ^4^ Department of Developmental and Educational Psychology, University of La Laguna, La Laguna, Canary Islands, Spain, ull.es; ^5^ Consortium for Biomedical Research in Epidemiology and Public Health (CIBERESP), Madrid, Spain, ciberesp.es; ^6^ Institute for Applied Microelectronics, University of Las Palmas de Gran Canaria, Las Palmas de Gran Canaria, Spain, ulpgc.es; ^7^ Canarian Health Research Institute Foundation (FIISC), Las Palmas de Gran Canaria, Spain; ^8^ Research Unit, University Hospital of Gran Canaria Dr. Negrin, Las Palmas de Gran Canaria, Spain; ^9^ Pediatrics Department, Insular-Maternal and Child University Hospital Complex of Las Palmas de Gran Canaria, Las Palmas de Gran Canaria, Spain; ^10^ Endocrinology and Nutrition Department, Insular-Maternal and Child University Hospital Complex of Las Palmas de Gran Canaria, Las Palmas de Gran Canaria, Spain; ^11^ Insular-Maternal and Child University Hospital Complex of Las Palmas de Gran Canaria, Las Palmas de Gran Canaria, Spain; ^12^ Pediatric Gastroenterology and Nutrition Unit, Insular-Maternal and Child University Hospital Complex of Las Palmas de Gran Canaria, CIBEROBN ISCIII, University of Las Palmas de Gran Canaria, Las Palmas de Gran Canaria, Spain, ulpgc.es

**Keywords:** children, multicomponent, obesity prevention, protocol, schools

## Abstract

This manuscript describes the design and baseline findings of the POI trial, a school‐based, cluster‐randomized intervention aimed at the prevention of childhood obesity in the Canary Islands, Spain. Childhood obesity prevalence in this region is among the highest in Europe, with 40.6% of children aged 6–9 years classified as overweight or obese. Early, multidisciplinary, school‐based interventions integrating nutrition and physical activity are therefore a public health priority.

**Methods:**

The POI trial is a cluster‐randomized controlled study conducted in primary schools in Gran Canaria, enrolling children aged 6–10 years. The intervention integrates nutrition education, physical activity promotion, and family engagement, supported by both paper‐based and digital educational materials. Teachers played a central role in intervention delivery within the classroom setting. Schools served as the unit of randomization and implementation. Baseline assessments included standardized anthropometric measurements, objectively measured physical activity, and selected metabolic parameters (fasting glucose and lipid profile). Dietary intake outcomes will be reported separately; this manuscript focuses on the nutrition education framework and baseline nutrition‐related metabolic indicators.

**Results:**

A total of 557 children from 13 schools were included (298 intervention participants from 5 schools and 259 controls from 8 schools). Significant differences in overweight and obesity prevalence were observed by school type, with the highest prevalence in public schools (48.6%), followed by partially subsidized (33.2%) and private schools (28.3%) (*p* = 0.001). Among participants with available blood samples (*n* = 237), elevated fasting glucose was detected in 21 children and hypercholesterolemia in 24 children.

**Conclusions:**

These baseline findings highlight substantial socioeconomic disparities in childhood overweight and obesity and demonstrate the presence of early metabolic risk in school‐aged children. The POI trial provides a scalable, school‐based model integrating nutrition education and physical activity for obesity prevention, with the potential to inform public health strategies in high‐prevalence settings.

**Trial Registration:** ClinicalTrials.gov identifier: NCT06607159

## 1. Introduction

Overweight and obesity are forms of malnutrition characterized by excess calorie intake, which significantly impact population health with cumulative effects over the life course. Obese youth exhibit elevated cardiovascular risk factors and are more likely to remain obese into adulthood [1]. Furthermore, they face increased risks of respiratory distress, fractures, hypertension, and diabetes mellitus. These physical health issues are often compounded by mental health challenges, social stigma, discrimination, and difficulties in relationships, all of which can substantially diminish their overall quality of life [2].

Childhood obesity has reached epidemic proportions globally. According to the World Health Organization (WHO), around 1 in 3 children aged 6–9 years in Europe live with overweight or obesity [3]. To address this issue, the WHO Regional Office for Europe established the Childhood Obesity Surveillance Initiative (COSI) in 2007 [4]. COSI aims to monitor trends in overweight and obesity among primary school children aged 6–9 years across Europe using standardized anthropometric measurements. The initiative has grown to include 45 participating countries to date.

In Spain, the ALADINO (Alimentación, Actividad física, Desarrollo Infantil y Obesidad) study is conducted as part of the COSI framework. It has undergone four rounds so far (2011, 2013, 2015. and 2019), providing nationally representative data on the weight status of Spanish children aged 6–9 years [5]. By 2019, ALADINO reported a combined excess weight prevalence of 40.6%, a modest decrease from 2011 [5]. However, obesity rates remained high and did not decline significantly over this period. The enKid study [6], conducted between 1998 and 2000, showed that the Canary Islands had one of the highest rates of overweight and obesity among Spanish children aged 6–13 years (prevalence of overweight/obesity of 32.8%). The ALADINO study confirmed the severity of the situation, placing the Canary Islands among the regions with the highest childhood obesity prevalence not just in Spain, but globally.

Intervention measures are more effective the earlier they are taken and the pediatric age is an ideal window of opportunity [7]. Recent reviews conclude that a multidisciplinary approach that takes into account a variety of factors including nutrition, physical activity, sleep time, and psychological intervention is more effective than interventions focused on only one area [8]. There is also strong evidence for school‐based interventions, with additional home or community‐based intervention components [9], with the added benefit that the introduction of nutrition education in schools has been shown to be cost‐effective [10].

Of special interest is the role of the socioeconomic status (SES) on obesity. Several studies in developed countries, including Spain, have shown the impact it has on its prevalence, highlighting the role parental income and education have on overweight and obesity rates [11, 12].

Given the alarming rates of obesity and overweight in our child population, we have developed an intervention strategy, based on a multidisciplinary, school‐centered approach, which addresses nutritional education, physical activity, behavioral intervention techniques, and tools that facilitate the learning of healthy lifestyle habits in children between 6 and 10 years of age. This article describes our design and implementation framework for a school‐based intervention on healthy lifestyle habits combining school, family, and student‐level components among children aged 6–10 years. It also describes the baseline characteristics and initial socioeconomic (SES) disparities found in overweight and obesity prevalence in our population.

## 2. Methodology

### 2.1. Study Design

This is a parallel, cluster‐randomized, controlled trial designed to evaluate a school‐based intervention for childhood obesity prevention in the Canary Islands. The study protocol was designed following SPIRIT guidelines. It was preregistered at AsPredicted.org, hosted by the University of Pennsylvania (registration No. 44205).

### 2.2. Participants and Randomization Process

Boys and girls aged 6–10 years (1^st^ to 4^th^ grades of primary school) from public, state‐subsidized, and private schools on the island of Gran Canaria, Canary Islands, Spain, were recruited. The selection of schools was based on convenience. After obtaining a list of schools from contacts with the local diabetic association and members of the team, the project was explained and schools were invited to participate. After obtaining the consent from several schools, the inclusion in both arms of the study was randomized with a 1:1 ratio (even though we performed cluster randomization, the aim was to have a 1:1 participant ratio, not a 1:1 classroom ratio), and the parents of the students in the aforementioned classes were invited to participate. Finally, those children whose parents consented to their participation were included. To randomize the participant schools, we employed a stratified cluster randomization approach. First, we categorized schools into three types: public, private, and subsidized. In Spain, type of school (public, subsidized, or private) is a strong indicator of socioeconomic background, as documented in previous national studies. This classification has therefore been used as a proxy for SES in the current analysis (each type of school represents a different SES, with a spectrum ranging from the lowest SES in the public schools, to the highest in the private ones [11]). Although randomization was stratified by school type (public, subsidized, and private) to account for socioeconomic differences, perfect balance between intervention and control groups was not achieved. This was due to variability in school sizes and the decision to prioritize a 1:1 ratio of participants rather than number of schools. This approach was chosen to preserve statistical power while maintaining the integrity of cluster randomization.

Within each category, we assigned a random number to each school and then ranked them from highest to lowest based on these numbers. Given that we knew the number of children in each class, we started allocating schools with the highest random numbers to either the intervention or control arm, continuing this process until the target number of children in each arm was reached. This approach ensured a 1:1 ratio of participants between arms while maintaining randomization integrity, even though it could result in an unequal number of schools per arm. To maintain cluster integrity and reduce contamination bias, all children from a given school were assigned to the same study arm—a methodological strength in school‐based interventions. Randomization was performed by the main author (YNM) using a stratified cluster randomization method in Microsoft Excel, assigning random numbers and stratifying by school type.

### 2.3. Intervention Protocol

The project for the prevention of childhood obesity in the Canary Islands aims to improve knowledge, attitudes, and behaviors related to healthy lifestyle habits in the pediatric age group through an educational and participatory approach (based on recommendations on diet [13, 14], physical activity [15, 16], leisure time devoted to screens [17], and hours of sleep [18]). One of our main goals was to develop cost‐free strategies and tools for schools. To achieve this, we decided to involve teachers as the primary drivers of change and schools as the implementation sites for the study protocol. To this end, the pediatric endocrinology and gastroenterology units of the Complejo Hospitalario Universitario Materno Infantil de Las Palmas de Gran Canaria have collaborated with the group specializing in “Learning Difficulties, Psycholinguistics, and Information and Communication Technologies (DEAP&NT)” of the University of La Laguna (https://viinv.ull.es/grupos/1119/), which includes psychologists, teachers, and pedagogues among its members.

As a result of this collaboration, a protocol for teaching healthy lifestyle habits has been developed, aimed at school‐age children (initially between 1^st^ and 4^th^ grades of primary school), to be implemented transversally in schools. This protocol takes into account three levels of action: school, family, and pupils.

a. Materials in paper format: these materials are mainly used in the classroom, although they can also be used at home. They have been designed according to the grade level and the level of competence of the students. These workbooks are based on the curricular contents linked to healthy lifestyle habits that should be worked on according to the school year as established in DECREE 89/2014, of 1 August, which establishes the organization and curriculum of primary education in the Autonomous Community of the Canary Islands [19]. Likewise, from an inclusive perspective, suitable materials have been designed for students with specific educational support needs (SEN) that make it difficult for them to access the written code (Figure 1(a)). While all levels included mentions of sleep hygiene, specific guided activities were only included at levels 0–1, based on age‐appropriateness and curricular alignment. Higher levels introduced sleep indirectly through discussions on lifestyle balance. The books can be found in the following link: https://www.fundacionmapfrecanarias.org/formacion-prevencion/actividades-didacticas/programa-obesidad-infantil-fmc/.
b. Digital materials: gamification has been proven to motivate and promote improvements in the nutrition of children [20]. For our intervention, 6 games have been designed in application format aimed at increasing daily physical activity time (2 games: 5 min of movement and active breaks), a balanced diet (2 games: healthy breakfasts and the cool recipe), and knowledge and reflection on healthy lifestyle habits (2 games: asking questions and my alien) (Figure 1(b)). The digital materials are embedded in a web platform that allows teachers to know the health status of the classroom based on the scores obtained by the students in each of the mini games. The games can be used in the classroom or at home, thus trying to facilitate parental interaction with the program (the digital materials were limited to a maximum of 30 min per day, with an emphasis on active learning and classroom‐based use, minimizing risk of screen overexposure. Additionally, all content was gamified to promote physical activity, not passive screen use. No minimum time limit was imposed for the study). It is important to emphasize that we will always work with the classroom score, emphasizing that health is everyone’s responsibility.

The family component of the intervention was primarily designed to reinforce school‐based learning through home engagement. Children were encouraged to use both paper‐based materials and digital tools outside the classroom, facilitating indirect parental involvement. While no formal parent training sessions were implemented, families had access to all materials and were encouraged to participate in activities, particularly those related to nutrition and lifestyle habits. This approach was intended to extend behavior change beyond the school environment while maintaining feasibility within a large‐scale intervention.

FIGURE 1Developed materials. (a) Printed material and (b) digital material.

**Figure:**
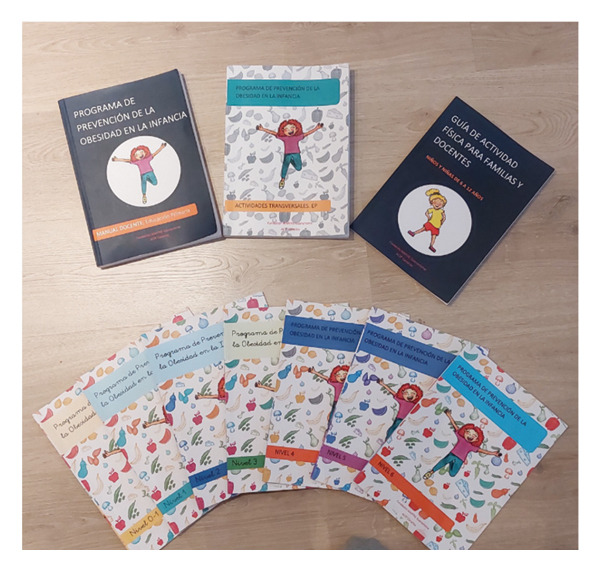
(a)

**Figure:**
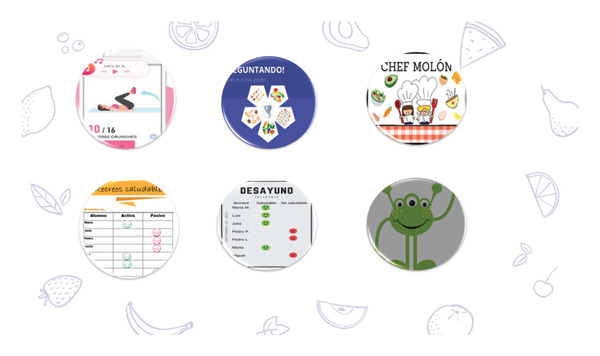
(b)

#### 2.3.1. Intervention Delivery and Implementation

The program was initiated with an evaluation phase (April–June 2022) and a teacher training phase (April–June 2022) before initiating the implementation over one academic year (September 2022 to June 2023) and integrated into routine classroom activities. Teachers were responsible for implementing the intervention components within their usual teaching schedule.

The paper‐based materials (workbooks) were aligned with the official primary education curriculum (Decree 89/2014) and designed to be incorporated into subjects such as natural sciences, physical education, and tutoring sessions. Teachers were encouraged to deliver activities on a regular basis throughout the school year, typically integrating them into weekly classroom sessions. While a standardized set of materials and objectives was provided, teachers retained flexibility to adapt the timing and pacing of activities according to classroom needs.

Digital materials consisted of six gamified applications targeting physical activity, nutrition, and healthy lifestyle knowledge. Teachers were encouraged to incorporate these tools periodically during classroom time and to promote their use at home. Although no strict minimum usage was imposed, digital exposure was limited to a maximum of 30 min per day, with an emphasis on active engagement rather than passive screen time.

The intervention followed a semistructured approach: core content and materials were standardized across schools, while allowing adaptability in delivery to enhance feasibility and real‐world applicability. Post‐intervention measurements were taken in April–June 2023 (will be reported separately). Table 1 summarizes the schedule of enrollment, interventions and assessments.

**TABLE 1:** SPIRIT schedule of enrollment, interventions, and assessments.

Study period	Activity	Enrollment (T0)	Baseline	Intervention	Post‐intervention
Enrollment	Informed consent	X			
Enrollment	Eligibility screening	X			
Assessments	Anthropometric measurements		X		X
Assessments	Physical activity (accelerometry)		X		X
Assessments	Body composition		X		X
Assessments	Metabolic parameters (glucose and lipids.)		X		X
Intervention	Multicomponent school‐based education			X	
Intervention	Teacher training	X	X	X	
Outcomes	Primary and secondary outcomes (e.g., BMI z‐score)				X

##### 2.3.1.1. Control Arm

The control group was measured at the same time as the intervention arm. No implementation was planned for the control schools.

#### 2.3.2. Implementation Fidelity and Adherence

In order to ensure fidelity of implementation of the intervention, Likert‐type scales for teacher self‐assessment and external observation were designed. These scales have 5 response options (0 = never to 4 = always), with a mean score ≥ 3 considered indicative of adequate fidelity. Teachers also recorded the number of intervention sessions delivered throughout the academic year, allowing quantification of exposure to the intervention. Adherence to the intervention was monitored through these logs and evaluation tools, enabling assessment of both the consistency and intensity of implementation across participating schools.

### 2.4. Outcome Measures

Our primary outcome is the change in body mass index (BMI). WHO growth curves were used to define overweight and obesity [21]. BMI was selected as the primary outcome due to its wide acceptance, ease of measurement, and comparability with national and international surveillance systems (e.g., WHO, ALADINO, and COSI). While BMI has limitations—notably its inability to distinguish fat mass from lean mass—it remains the most standardized and feasible anthropometric measure for large‐scale interventions in pediatric populations. Previous trials and systematic reviews comment on the use of BMI *z*‐score as an outcome in school‐based obesity prevention programs [22–24]. In our study, BMI was complemented by additional measures such as body fat percentage, waist circumference, and metabolic markers, allowing for a more nuanced understanding of body composition and health outcomes.

Secondary outcomes are changes in triponderal mass index (TMI), weight, body fat percentage, waist circumference, physical activity, and metabolic parameters.

Anthropometric measurements included weight, height, BMI, TMI, body fat percentage, and waist and hip circumferences. Healthcare personnel conducted the measurements at the schools attended by the children. Standardized conditions were adapted from the 2019 ALADINO study in the Canary Islands [25]. The WHO definition of overweight and obesity was used to define overweight and obesity: overweight and obesity are defined as “BMI‐for‐age and weight‐for‐height with more than one and two standard deviations (SDs), respectively, above the median of the WHO child growth standards” for > 5 years. The measurements were taken using a Tanita model DC‐360 impedance scale (Tokyo, Japan) for weight and body fat percentage, a portable TANITA stadiometer (Leicester model) for height, and a flexible, nonstretchable anthropometric measuring tape for waist and hip circumference. Nutritional ultrasound was performed using an L6C transducer (Microcaya) on a subset of children who provided consent for blood sample analysis. The ultrasound measurements focused on quantifying subcutaneous abdominal fat and preperitoneal fat tissue thickness. While the results are not presented in this manuscript, these measurements were collected to assess body composition and fat distribution in the context of obesity prevention.

Physical activity was objectively assessed in a random sample of 74 participants using the GENEActiv accelerometer (Activinsights Ltd., Kimbolton, United Kingdom). The participants were instructed to wear the accelerometer on their dominant wrist for 8 consecutive days and nights, including both weekdays and weekend days. Data were processed using RStudio software version 2022.07.2 + 576 (RStudio Team [2022]). RStudio: Integrated Development Environment for R. Boston, MA: RStudio, PBC) and the accelerometer manufacturer provided the processing algorithm for data segmentation and classification. The GENEActiv accelerometer is a triaxial accelerometer that measures acceleration in three orthogonal planes (*x*, *y*, *z*), which was configured at a sampling rate of 100 Hz. From the raw acceleration data, the processing algorithm provides activity intensity metrics, including time spent in sedentary, light, moderate, and vigorous physical activity. Classification is based on the vector magnitude of the acceleration signals. Specifically, the software calculates the Euclidean norm minus one (ENMO) of the acceleration signals, which provides a measure of the dynamic acceleration. For this study, only data relating to moderate and vigorous physical activity were considered. The number of participants was chosen based on the number of available accelerometers and available time for its use. We chose participants aged 8–9 years, in order to facilitate comparison with the PASOS 2019 study [26], the largest national study performed to date in Spain looking at the physical activity of children. We randomized the number of participants based on the type of school and arm (intervention vs control), aiming for a 1:1 ratio: 38 participants came from public schools and 36 from private and partially subsidized centers. A total of 37 participated in the intervention arm and 37 in the control arm.

Accelerometry data were analyzed separately for school days and nonschool days. For school days, we further stratified the analysis into two time periods: school hours (08:30 to 16:00) and after‐school hours (16:01 to 21:00), to distinguish between physical activity performed during school time and outside school hours. Minimum use included full use of the accelerometer during the study period (24 h/d for 8 days).

Metabolic parameters: fasting peripheral blood samples were taken from consenting participants (whose families had also given written consent) in two tubes with ethylenediaminetetraacetic acid (EDTA) and no additives, respectively. Samples were centrifuged and plasma and serum were separated.

The following tests were obtained using standardized, automatic methods: blood count was assessed in total blood, and C‐reactive protein (CRP), glucose, creatinine, transaminases, total and HDL cholesterol, triglycerides, and insulin were measured in serum. Insulin was measured with a chemiluminescent immunoassay using paramagnetic particle technology on the DXI 800 analyzer (Beckman Coulter, Brea, CA). HOMA index was calculated with the following formula: HOMA‐IR = (fasting glucose (mg/dL)  × fasting insulin (μUI/mL))/405. Glucose metabolism disorders were classified according to ADA criteria [27]. Impaired fasting glucose was defined as a fasting glucose value of 100–125 mg/dL. Dyslipidemia was diagnosed based on international recommendations [28]. Hypercholesterolemia was defined as total cholesterol > 200 mg/dL.

Other variables: although the intervention addressed behaviors such as eating habits, screen time, and sleep hygiene, these were not quantitatively assessed at baseline due to time and resource limitations. Their inclusion in subsequent evaluations is planned.

It is worth mentioning that post‐intervention results will not be presented here.

### 2.5. Statistical Analysis

Sample size (286 kids in each arm of the study) was estimated using the “two independent means” approach, assuming an effect size of 0.75 BMI units (SD = 1.5), 80% power, *α* = 0.05 (two‐tailed), and an expected attrition rate of 15%. The effect size of 0.75 BMI units was selected based on a balance between clinical relevance and the practical limitations of our study. Given that our sample was limited to children who volunteered to participate, a larger sample was not feasible. Therefore, we opted for an effect size of 0.75 BMI units, which we considered both clinically meaningful in the context of pediatric obesity prevention [22, 24] and achievable within the constraints of our available sample size. Statistical analyses were performed using STATA version 14MP (StataCorp LLC, College Station, TX, USA).

Although the sample size calculation was initially performed at the individual level using a two independent means approach, we acknowledge that the cluster‐randomized design requires consideration of intracluster correlation (ICC). The absence of this adjustment may have led to an overestimation of statistical power, and this is recognized as a limitation. To appropriately account for clustering, all outcome analyses will be conducted using statistical models that consider the hierarchical structure of the data, with children nested within schools. Specifically, mixed‐effects regression models (or cluster‐robust standard errors) will be used to adjust for school‐level clustering.

In the descriptive statistical study, means, minimum and maximum values, and SD were calculated for quantitative variables. For qualitative variables, counts and proportions are presented. The 95% confidence interval for mean values and proportions was calculated. The hypothesis test for comparing proportions was used to verify the difference between proportions and the Student’s *t*‐test or Mann–Whitney test (if the distribution was not normal) was used to analyze differences between means of two samples. In the case of more than two samples, the tests applied were ANOVA or Kruskal–Wallis (non‐normal samples). Significant differences were considered when *p* < 0.05. Pearson correlation coefficient was used to evaluate the existence of correlation between average moderate/vigorous physical activity per day and BMI. Outcome assessors were blinded to school allocations to ensure unbiased evaluation of results.

At the end of the study, the primary and secondary outcomes will be reassessed. The primary analysis will be conducted according to the intention‐to‐treat (ITT) principle, where all randomized participants will be analyzed according to their assigned treatment group, regardless of adherence or withdrawal. Missing data will be handled using multiple imputation techniques. The treatment effect for each outcome will be estimated using appropriate statistical models, adjusting for baseline values of the outcome and other relevant covariates. Multivariate linear and logistic regression models will be used to evaluate intervention effects, adjusting for baseline covariates such as age, sex, and school type. Interaction terms will explore effect modification by SES and baseline BMI category. All multivariate analyses will adjust for school type as a proxy for SES, given its known association with childhood obesity and its imbalance between study arms. No changes to the methodology were implemented after trial commencement.

## 3. Results

A total of 557 children from 13 schools participated in the study, 298 in the intervention group (from 5 schools) and 259 in the control group (from 8 schools). As anticipated in our methodology, achieving a 1:1 ratio of participants resulted in an unequal number of schools between the intervention and control groups due to variations in class sizes. This outcome was consistent with our randomization protocol, which prioritized participant balance over school number balance.

### 3.1. Anthropometry

Table 2 shows the characteristics of the participants.

**TABLE 2:** Anthropometric characteristics in the pre‐intervention situation.

	Control (*N* = 259)			Intervention (*N* = 298)			*p*‐values
	*N*	Mean (%)	SD	*N*	Mean (%)	SD	
School type	8			5			
Public	87	33.6%	n/a	121	40.6%	n/a	0.063
Partially subsidized	120	46.3%	n/a	109	36.6%	n/a	
Private	52	20.1%	n/a	68	22.8%	n/a	
Sex^∗^ (girls)	155	59.8%	n/a	152	51%	n/a	0.036^∗^
Age^∗^ (years)		8.1	1.3		7.6	1.2	0.000^∗^
Height^∗^ (cm)		133.2	9.8		130.7	8.5	0.002^∗^
Weight (kg)		31.8	9.7		30.6	8.5	0.125
Body mass index (kg/m^2^)		17.6	3.4		17.6	3.2	0.957
Weight *z*‐score							
Normal (0)	170	65.6%	n/a	176	59.1%	n/a	0.245
Overweight (+1)	47	18.2%	n/a	60	20.1%	n/a	
Obesity (+2)	42	16.2%	n/a	62	20.8	n/a	
Triponderal body index		13.2 kg/m^3^	2.2		13.5 kg/m^3^	2.1	0.129
% of body fat		21.8%	7.1		22.8%	6.8	0.092
Waist circumference (cm)		61.4 cm	10.1		59.9 cm	9.5	0.050^†^

Abbreviation: SD, standard deviation.

^†^Kruskal–Wallis.

^∗^ *p*‐value < 0.05.

Both groups are comparable in weight, BMI, TMI, weight z‐score, and body fat percentage, with slight differences in age, sex distribution, and height.

The overall rate of overweight and obesity in our sample was 37.9%. When assessing the distribution of overweight and obesity among the different schools (public, subsidized, and private), we found a significantly higher proportion of overweight and obesity in public schools than in subsidized schools and a higher proportion in subsidized schools than in private schools (*p* = 0.001, Table 3).

**TABLE 3:** Percentage of overweight and obesity by type of school.

	Type of school			
	Public	Partially subsidized	Private	Total
Adequate BMI, *N* (%)	107 (51.4)	153 (66.8)	86 (71.7)	346 (62.1)
Overweight, *N* (%)	46 (22.1)	41 (17.9)	20 (16.7)	107 (19.2)
Obesity, *N* (%)	55 (26.4)	35 (15.3)	14 (11.7)	104 (18.7)
Overweight + obesity (%)	48.6%	33.2%	28.3%	37.9%
Total (N)	208	229	120	557

*Note:* Pearson chi‐square: *x* ^2^(4) = 19.1993; *p* = 0.001.

Abbreviation: BMI, body mass index.

### 3.2. Metabolic Parameters

With regard to the blood tests carried out, 237 participants consented to their extraction: 109 in the intervention group and 128 in the control group. There were slight significant differences in glucose, total cholesterol, and LDL cholesterol levels. Blood tests detected 21 cases of impaired fasting blood glucose and 24 cases of hypercholesterolemia. Table 4 summarizes the results.

**TABLE 4:** Pre‐intervention analytical characteristics.

	Control (*N* = 109)				Intervention (*N* = 128)				*p*‐values
	Mean (%)	SD	Min.	Max.	Mean (%)	SD	Min.	Max.	
Glucose^∗^ (mg/dL)	90.1	6.9	72	114	93.1	6.9	77	114	0.0023^†^ ^∗^
Triglycerides (mg/dL)	62	28	26	211	57.6	25.4	29	251	0.2156
Total cholesterol^∗^ (mg/dL)	164.5	28.8	103	242	173.7	27.8	109	295	0.0266^†^ ^∗^
LDL cholesterol ^∗^ (mg/dL)	88.1	22	32	143.3	98.2	23.9	54.8	210.5	0.0026^†^ ^∗^
HDL cholesterol (mg/dL)	64	13.1	38.7	96.7	64	11.1	44.1	91.4	0.9782
Urea (mg/dL)	26.4	5.7	16	45	27.7	5.2	17	41	0.0642
GOT (AST) (UI/L)	32.6	7.1	19.2	68.9	33.7	6.5	21.4	58.4	0.1778
GPT (ALT) (UI/L)	18.9	8	9.3	68.4	18.7	5.2	9.7	38	0.7845
Hemoglobin (g/dL)	13.3	0.8	10.3	15.5	13.4	0.7	11.6	15.9	0.1424
Creatinine (mg/dL)	0.5	0.07	0.3	0.6	0.5	0.06	0.3	0.6	0.6317
CRP (mg/dL)	0.1	0.2	0.02	1	0.1	0.2	0.02	1	0.5331
Insulin (μUI/mL)	5.7	4.6	0.7	32.5	6.7	7.6	1.2	53.9	0.2915
HOMA index	1.3	1.2	0.1	8.7	1.6	1.9	0.2	15.2	0.1665^†^

*Note:* Min, minimum; Max, maximum.

Abbreviations: CRP, C‐reactive protein; HDL, high density lipoprotein; LDL, low density lipoprotein; SD, standard deviation.

^†^Kruskal–Wallis.

^∗^ *p*‐value < 0.05.

### 3.3. Physical Activity

Daily moderate/vigorous activity was, on average, 4.2 ± 1.2 h (252 ± 72 min), with significant differences between school and nonschool days (*p* < 0.001) (Figure 2(a)), and between morning (8:30–16 h) and afternoon (16:00 to 21:00 h) intervals (*p* < 0.001) (Figure 2(b)). We found statistically significant differences in moderate/vigorous activity during the school day mornings and afternoons among center types (*p* < 0.05) (Figures 3(a) and 3(b)). No significant differences were found between control and intervention groups (*p* = 0.74) or between boys and girls (*p* = 0.73). We did find a significant, inverse correlation between the amount of moderate/vigorous physical activity during school days and the BMI of the subjects (*r* = −0.26; *p* = 0.02).

FIGURE 2Average moderate/vigorous activity time in minutes per day. (a) During school and nonschool days and (b) during school day mornings and afternoons.

**Figure:**
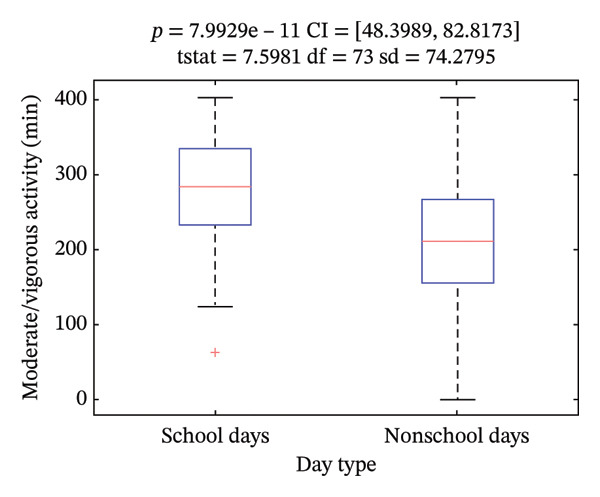
(a)

**Figure:**
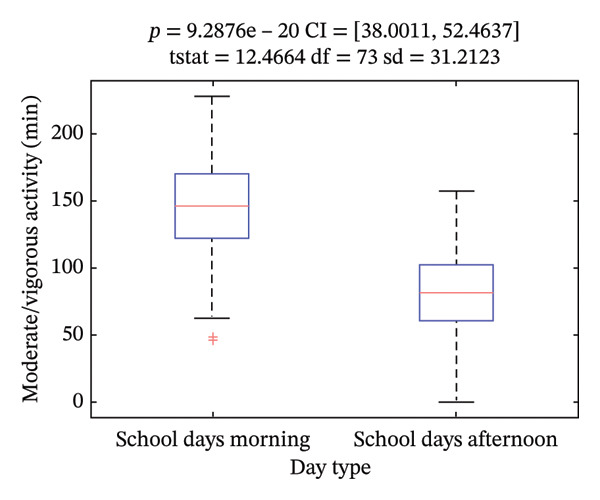
(b)

FIGURE 3Average moderate/vigorous activity time in minutes per day during school day mornings and afternoons. (a) Average moderate/vigorous activity time in minutes per day during school day and (b) average moderate/vigorous activity time in minutes per day during school day afternoons. Box plot and statistical analysis (*p*‐value). Center type: 1 (public); 2 (partially subsidized); and 3 (private).

**Figure:**
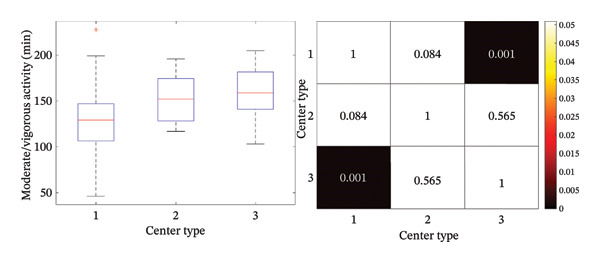
(a)

**Figure:**
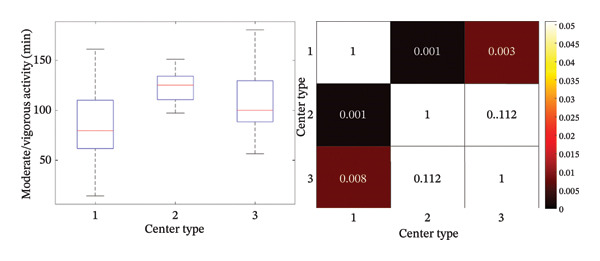
(b)

## 4. Discussion

This study describes the design of a randomized, controlled, parallel clinical trial assessing the impact of an educational intervention on the prevalence of obesity in children, as well as the baseline description of the study population. School type was used as a proxy for SES. Socioeconomic disparities were clearly present in our measurements, with a wide gap in overweight and obesity rates between public (48.6%) and private schools (28.3%). Also, our study highlights the early onset of dysglycemia and dyslipidemia in our population, emphasizing the need for targeted programs for at risk population.

Some of the strengths of our project include the development of the program in collaboration with educational specialists, the inclusion of components related to nutrition, physical education, sleep, and overall health, as well as the presence of both school‐ and home‐based areas of intervention, all of which follow current recommendations emphasizing the need for multilevel strategies. Also, our program has been designed to be implemented at no cost for the school system (all the cost for the development of the program has already been paid for). It is scalable, low‐cost, teacher‐led, and a digital‐analog hybrid. The lack of significant baseline differences in weight, BMI, and body fat percentage between intervention and control groups supports the comparability of both arms, reinforcing the internal validity of the subsequent evaluation.

International organizations like the WHO or the American Heart Association recommend promoting cardiovascular health in children by maintaining adequate BMI, optimal physical activity, and a balanced diet [29]. Current evidence supports that the ideal components for an obesity intervention program should include at least a combination of nutritional and physical education components [8, 30], even though sometimes the effects are small [31]. Other lifestyle factors like sleep or television time have also been shown to be related to an increased risk of overweight and obesity [32]. The earlier age groups (5–11 years) provide a clear window of opportunity, since the effect of these interventions is likely smaller as they progress into adolescence [33]. Interventions in school have proven effective [9], even with simple measures as targeting the promotion of drinking water vs other sources of sugar‐rich drinks [34] or increasing physical activity [35] (even with small increases like 20 min/day [36]).

### 4.1. Obesity Prevalence and Disparities Among Schools

With regard to the evolution of overweight and obesity in Spain in the last decade, data from the COSI initiative (ALADINO in Spain) show an increase between 2011 and 2015–17, with subsequent stabilization. The PASOS study [26], conducted in 2019–2020, assessed the nutritional status of Spanish children aged 8–16 years. The results showed that 23.3% were overweight and 17.3% were obese [37], further supporting the decreasing trend observed in ALADINO. However, other studies suggest an ongoing increasing trend [38]. In any case, obesity rates remain high in our country and remain a primary area for intervention in child´s health. At 37.9%, our rate is slightly lower than those described by PASOS and ALADINO, but that is probably due to the overrepresentation of private schools in our sample.

Our findings reveal significant disparities in overweight and obesity rates among different school types, with public schools showing the highest prevalence (48.6%) compared to partially subsidized (33.2%) and private schools (28.3%). This socioeconomic gradient in obesity is consistent with previous studies in Spain and other developed countries [11, 12, 39]. The SES was a clear factor influencing overweight and obesity rates. These disparities were also present in the PASOS study, with the influence of parental education and income in overweight and obesity rates, with reported increased rates in lower SES [11]. Also, further highlighting the uneven distribution of overweight and obesity among different SES strata, the PASOS study showed that the stabilization of overweight and obesity rates described in the last years by ALADINO is at the expense of the higher socioeconomic levels.

Importantly, our results also highlight the early onset of metabolic complications in this population. We detected cases of elevated fasting blood glucose (21 cases) and hypercholesterolemia (24 cases) across both groups. Childhood obesity is associated with increased risk of type 2 diabetes, dyslipidemia, hypertension, and metabolic syndrome [2]. Longitudinal studies have found childhood obesity and cardiovascular risk factors track into adulthood, with obese children having a higher risk of becoming obese adults [40].

### 4.2. Physical Activity

Regarding physical activity in our population, it is worth mentioning that we found significant disparities between the physical activity performed during school mornings and afternoons among centers, with more hours of moderate/vigorous physical activity in private and partially subsidized centers compared to public schools. Morning differences are explained by increased physical activity during school hours (increased recess or directed physical education/activities). Afternoon differences might be explained by more after‐school programs in private and partially subsidized centers or by SES differences playing a role in the decision to enroll the participating children in after‐school activities. This difference could partially explain the different prevalence of overweight and obesity between centers.

It is noteworthy that the average amount of physical activity observed in our study, at 4.2 h per day, substantially exceeds the WHO’s recommendation of 60 min daily. Our results differ from those reported in the PASOS 2019 study by Gómez et al. [26], which utilized the ActiGraph wGT3X‐BT accelerometer (Pensacola, FL, USA) in a broader Spanish population. They reported approximately 2.5 h as the mean time spent on moderate/vigorous physical activity for children aged 8–9 years. The 68% higher activity levels in our study warrant careful consideration.

Several factors may contribute to this discrepancy:

(1) Accelerometer differences: while GENEActiv and ActiGraph accelerometers have been reported to correlate well, studies have shown a 10%–15% difference in the number of minutes reported [41, 42]. The GENEActiv tends to measure slightly higher accelerations compared to the ActiGraph, which could partially explain our higher activity measurements; (2) data processing methods: the equivalence of activity outcomes between different accelerometers can be influenced by the data processing techniques employed; (3) regional variations: the Canary Islands’ unique geographical and cultural context may contribute to genuine differences in physical activity levels compared to mainland Spain. Factors such as climate, outdoor recreational opportunities, and local lifestyle habits could play a role; (4) temporal changes: given that our study was conducted after the PASOS 2019 study, there’s a possibility of temporal shifts in activity patterns, potentially influenced by public health initiatives or societal changes; and (5) sample characteristics: differences in the SES, urban/rural distribution, or other demographic factors between our sample and the PASOS study population could contribute to the observed disparity.

Future research should aim to standardize accelerometry protocols across studies to facilitate more direct comparisons. Additionally, investigating the specific factors contributing to higher activity levels in the Canary Islands could provide valuable insights for public health interventions in other regions. Despite these differences, both studies highlight the importance of objective physical activity measurement in pediatric populations and contribute to our understanding of activity patterns in Spanish children.

One of the limitations of our study is the relatively low number of subjects. Also, private schools are overrepresented in our sample, which is probably reflected in the overweight and obesity prevalence rates (37.9%), which are somewhat lower than those published by recent studies in our community (around 44%) [5]. The distribution of public vs. private schools was not balanced between study groups, which could introduce residual confounding related to socioeconomic differences. While randomization was cluster‐based, this imbalance will be considered in adjusted analyses and interpreted cautiously in future publications. A higher number of public schools, better representing the low SES population in our island, would better reflect the current needs and benefits from such programs in our population. The nature of our school‐based intervention made complete blinding challenging. Our randomization method, while stratified to ensure balance across school types, did not include mechanisms for allocation concealment. Consequently, it was not possible to blind schools, participants, or on‐site researchers to the intervention assignment once the study commenced. This potential lack of blinding could have introduced bias, particularly in self‐reported outcomes or in the behavior of participants and school staff who were aware of their allocation. While school type serves as a reliable SES proxy, future analyses will explore the feasibility of incorporating area‐level census data (e.g., average household income or parental education by catchment zone) to support SES adjustment in outcome models. Lastly, although incidence measures are optimal for evaluating the effectiveness of preventive interventions, we anticipate that a follow‐up period longer than 12 months may be required to observe meaningful shifts in the incidence of overweight and obesity in this population. Thus, for the short‐term evaluation, changes in continuous anthropometric indicators will be prioritized.

To mitigate these concerns, we implemented several strategies. First, we used objective measures where possible to reduce reporting bias. Second, outcome assessors were blinded to school allocations to ensure unbiased evaluation of results. Third, we standardized intervention delivery across schools to minimize variability. Despite these efforts, we cannot rule out the possibility that awareness of group assignment may have influenced our results to some degree. Future studies might consider employing a wait‐list control design or developing interventions with less visible differences between arms to enhance blinding. Additionally, using a centralized randomization system could improve allocation concealment in similar school‐based trials.

In conclusion, our findings underscore the urgent need for targeted interventions to address the high rates of childhood obesity and associated metabolic complications in the Canary Islands, particularly in lower socioeconomic groups. The proposed multidisciplinary, school‐based approach has the potential to instill healthy behaviors and reduce obesity‐related risks in this vulnerable population. Further research is needed to evaluate the long‐term impact of such interventions on childhood and adult health outcomes.

## Author Contributions

Yeray Nóvoa‐Medina participated in the conception, design, planning, data acquisition, development of materials used in the intervention, analysis, and interpretation of the data, and development of the initial draft and subsequent versions of the manuscript; Sara del Cristo Leon and Eugenio Jiménez participated in the conception, design, development of materials used in the intervention, and development and revision of subsequent versions of the manuscript; Marta Barreiro‐Bautista participated in data acquisition and development of subsequent versions of the manuscript; Verónica Batista‐Dávila participated in data acquisition, analysis and interpretation of the data, and development of subsequent versions of the manuscript; Svetlana Pavlovic collaborated in the planning, data acquisition, and development of subsequent versions of the manuscript; Yaiza García‐Delgado, Sara López‐López, and the Gran Canarian Diabetes and Obesity Research Group participated in data acquisition and development of subsequent versions of the manuscript; Himar Fabelo collaborated in data acquisition, accelerometry analysis, and interpretation and development of subsequent versions of the manuscript; Ana María Wägner and Luis Peña‐Quintana participated in conception, design, planning, development of materials used in the intervention, and development of the initial draft and subsequent versions of the manuscript.

## Funding

This research was funded by Fundación Mapfre Guanarteme (project number OA1/129).

## Disclosure

The funders had no role in the design of the study; in the collection, analyses, or interpretation of the data; in the writing of the manuscript; or in the decision to publish the results. All authors approved the final version of the manuscript and agreed both to be personally accountable for their own contributions and to ensure that questions related to the accuracy or integrity of any part of the work, even ones in which they were not personally involved, are appropriately investigated, resolved, and resolution documented in the literature.

## Ethics Statement

The study was conducted according to the guidelines of the Declaration of Helsinki and was approved by the Ethics Committee of Hospital Universitario de Gran Canaria Dr. Negrín (protocol code 2020‐356‐1; approved on October 2^nd^, 2020). Informed consent was obtained from all subjects involved in the study. Written informed consent was obtained from a parent or guardian for all participants.

## Consent

Please see the Ethics Statement.

## Conflicts of Interest

The authors declare no conflicts of interest.

## Data Availability

The data that support the findings of this study are available on request from the corresponding author. The data are not publicly available due to privacy or ethical restrictions.
